# Development and validation of a porcine organ model for training in essential laparoscopic surgical skills

**DOI:** 10.1111/iju.14315

**Published:** 2020-08-03

**Authors:** Madoka Higuchi, Takashige Abe, Kiyohiko Hotta, Ken Morita, Haruka Miyata, Jun Furumido, Naoya Iwahara, Masafumi Kon, Takahiro Osawa, Ryuji Matsumoto, Hiroshi Kikuchi, Yo Kurashima, Sachiyo Murai, Abdullatif Aydin, Nicholas Raison, Kamran Ahmed, Muhammad Shamim Khan, Prokar Dasgupta, Nobuo Shinohara

**Affiliations:** ^1^ Department of Urology Hokkaido University Graduate School of Medicine Sapporo Hokkaido Japan; ^2^ Department of Urology Kushiro City General Hospital Kushiro Hokkaido Japan; ^3^ Hokkaido University Clinical Simulation Center Hokkaido University Graduate School of Medicine Sapporo Hokkaido Japan; ^4^ Division of Transplantation, Immunology and Mucosal Biology Faculty of Life Sciences and Medicine King’s College London London UK

**Keywords:** animal organs, laparoscopic surgery, simulation, surgical education, wet lab training

## Abstract

**Objectives:**

To develop a wet laboratory training model for learning core laparoscopic surgical skills and evaluating learners’ competency level outside the operating room.

**Methods:**

Participants completed three tasks (task 1: tissue dissection around the aorta; task 2: tissue dissection and division of the renal artery; task 3: renal parenchymal closure). Each performance was video recorded and subsequently evaluated by two experts, according to the Global Operative Assessment of Laparoscopic Skills and task‐specific metrics that we developed (Assessment Sheet of Laparoscopic Skills in Wet Lab score). Mean scores were used for analyses. The subjective mental workload was also assessed (NASA Task Load Index).

**Results:**

The 54 participants included 32 urologists, eight young trainees and 14 medical students. A total of 13 participants were categorized as experts (≥50 laparoscopic surgeries), eight as intermediates (10–49) and 33 as novices (0–9). There were significant differences in the Global Operative Assessment of Laparoscopic Skills and Assessment Sheet of Laparoscopic Skills in Wet Lab scores among the three groups in all three tasks. Higher NASA Task Load Index scores were observed in novices, and there were significant differences in tasks 1 (Kruskal–Wallis test, *P* = 0.0004) and 2 (*P* = 0.0002), and marginal differences in task 3 (*P* = 0.0745) among the three groups.

**Conclusions:**

Our training model has good construct validity, and differences in the NASA Task Load Index score reflect previous laparoscopic surgical experiences. Our findings show the ability to assess both laparoscopic surgical skills and mental workloads, which could help educators comprehend trainees’ level outside the operating room. Given the decreasing opportunity to carry out pure laparoscopic surgeries because of the dissemination of robotic surgery, especially in urology, our model can offer practical training opportunities.

**Abbreviations & Acronyms**
ALL: Assessment Sheet of Laparoscopic Skills in Wet Lab
ESSQ: endoscopic surgical skill qualification
GOALS: Global Operative Assessment of Laparoscopic Skills
ICC: interclass coefficient
NASA‐TLX: NASA Task Load Index
ROC: receiver operating characteristic
VR: virtual reality

## Introduction

Because of precise movements of instruments based on tremor filtration and motion scaling, high‐definition 3‐D vision and better ergonomics of the surgeon’s console, robot‐assisted surgery has been widely utilized in various surgical specialties.^1^, ^2^, ^3^, ^4^, ^5^ In urology, the numbers of robot‐assisted prostatectomies, cystectomies and partial nephrectomies are continuously increasing, resulting in decreasing opportunities for young trainees to carry out pure laparoscopic procedures.^1^, ^6^, ^7^ This trend will continue, but we consider that essential laparoscopic surgical skills are still necessary; for example, to cope with intra‐abdominal adhesion at the time of port placement or robot‐malfunction, and when taking an assistant’s role during robotic surgery. For example, in robot‐assisted radical prostatectomy, an assistant is required to place Hem‐o‐lok clips on vascular pedicles derived from neurovascular bundles, or to deliver the needles for vesicourethral anastomosis. In addition, several studies have shown that the acquisition of essential laparoscopic skills made the learning of robotic surgical skills easier. For example, Angell *et al*. reported that in a laparoscopic and naïve cohort (medical students), intensive laparoscopic training in basic laparoscopic skills reduced the time required to carry out the task (pattern cutting) robotically, as well as reduced the number of errors.^8^

In general, the box trainer is the most frequently utilized to learn laparoscopic surgical skills because of its low cost and easy accessibility.^9^ However, non‐biological materials might not be ideal to learn tissue dissection skills. In addition, the box trainer might be boring, especially for young trainees. In contrast, animal lab training could provide more practical training, but at a much higher cost, and the fact that anatomy differs from that of humans and animal welfare should be considered. A VR simulator provides a customized curriculum without the burden of any preparation or disposal after training, and data on objective metrics are fed back to trainees onsite. However, previous studies showed that residents preferred live animals or animal tissue wet lab as simulation tools, rather than a VR simulator.^10^, ^11^

Since June 2017, aiming to develop a practical training program for essential laparoscopic surgical skills, we started the current wet lab training using porcine cadaveric organs. In the present study, we evaluated the usefulness of our training model to evaluate laparoscopic surgical skill levels and mental workloads of participants outside operating theaters.

## Methods

The institutional review board approved the present study (No. 017‐0043). In 2016, we held several preliminary training sessions, and developed three training drills described in the next paragraph. Since 2017, we have regularly held the present wet lab training at Hokkaido University Clinical Simulation Center, usually on weekends, every 3–4 months. Written informed consent was obtained regarding the research use of the data.

### Training drills

#### Task 1

**Fig. 1:**
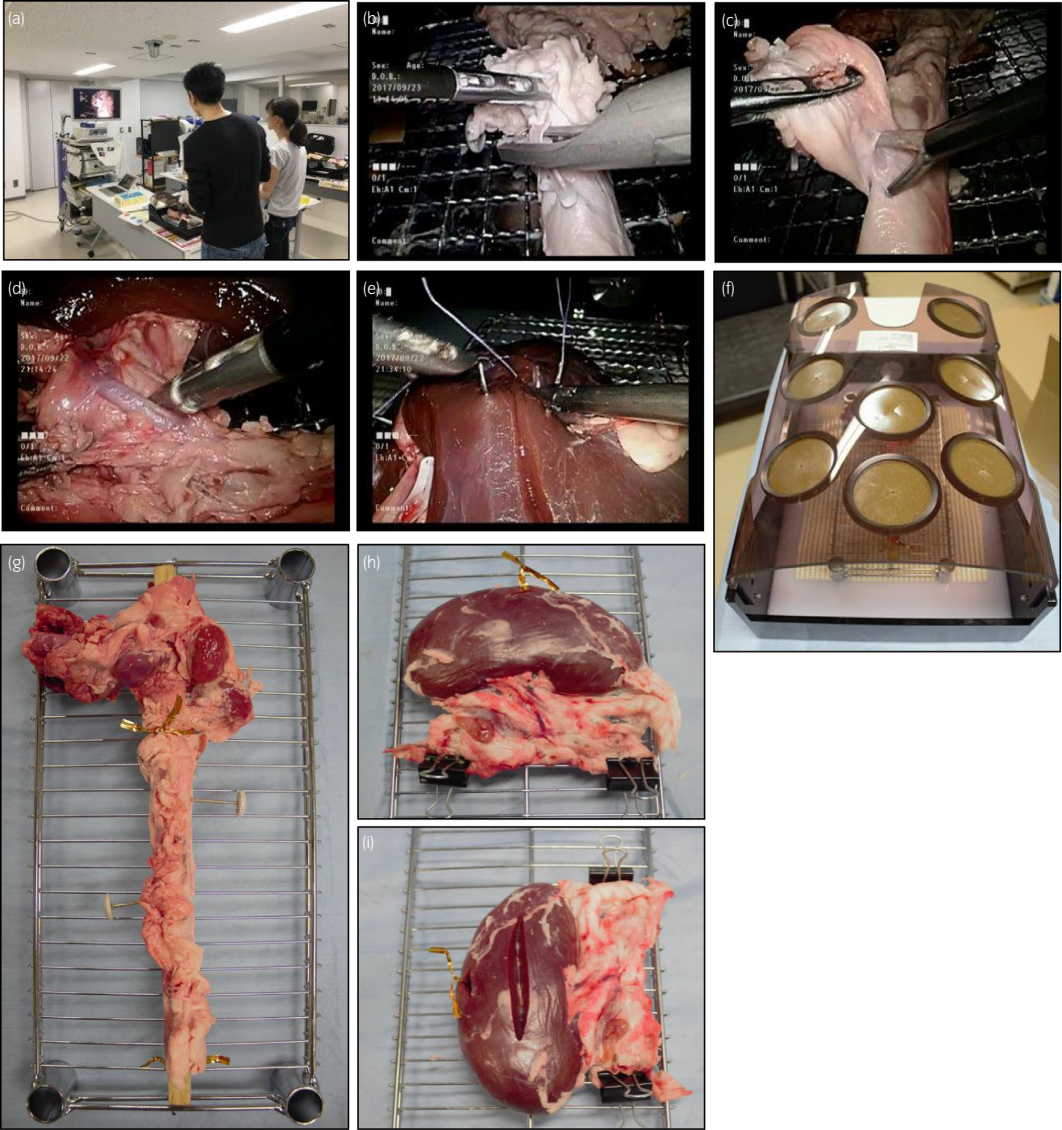
Photographs of the simulation training. (a) Training view. Candidates were informed of training slots, prepared on the Doodle website (calendar tool for time management and coordinating meetings), via email. Thereafter, participants voluntarily booked convenient slots according to their schedules, and participated in the training. (b,c) Task 1 (tissue dissection around aorta). (d) Task 2 (tissue dissection and divide renal artery). In tasks 1 and 2, laparoscopic scissors (Scissors Metzenbaum; Olympus, Tokyo, Japan), laparoscopic grasping forceps (CLICKline CROCE‐OLMI Grasping Forceps; Karl Storz, Tokyo, Japan) and a laparoscopic clip applier (Hem‐o‐lok Endoscopic Appliers Large; Teleflex, Tokyo, Japan) were used. (e) Task 3 (renal parenchymal closure). In task 3, laparoscopic needle holders were used (KOH Macro Needle Holder, ratchet position right, jaws curved to left, and KOH Macro Needle Holder, ratchet position left, jaws curved to right; Karl Storz). (f) Box trainer. (g) Setting of aorta in task 1. (h) Setting of kidney in task 2. (i) Setting of kidney in task 3.

This task is expected to help young trainees learn laparoscopic dissection skills around vessels and Hem‐o‐lok clip application. Participants are required to remove lymph nodes around the aorta, and divide encountered mesenteric vessels after applying Hem‐o‐lok clips (Fig. 1b,c; Video S1). Usually, five to seven mesenteric vessels were divided during the task.

#### Task 2

Task 2 was also designed to help learn laparoscopic dissection skills around vessels. With the use of the kidney pedicle, we aimed to mimic laparoscopic nephrectomy (Fig. 1d).

#### Task 3

Task 3 provides training in laparoscopic suturing mimicking renal parenchymal closure after partial nephrectomy. Participants are given a 25‐cm 2‐0 CT‐1 Vicryl thread (Ethicon/Johnson & Johnson, Tokyo, Japan), and are required to pass the needle from right to left through the kidney parenchyma, completing three square single‐throw knots, using a slip‐knot technique between the second and third knots, at three different sites on a kidney (Fig. 1e).

If participants had problems with simulation, especially medical students, they were verbally guided through each step by a scopist. In all three tasks, porcine cadaveric organs were placed in a box trainer (Endowork Pro II; Kyoto Kagaku, Kyoto, Japan; Fig. 1f). Porcine organs were purchased from a commercial vendor. In task 1, a 27‐cm long and 1.0‐cm wide stick made of balsawood was placed in the lumen of the aorta to stabilize its cylindrical shape (Fig. 1g). Two pins were pushed through the aorta to prevent rotation. In task 2, the porcine kidney was placed on its side and laterally to allow the renal hilum to face the trainee (Fig. 1h). The kidney pedicle was fixed with two clips. In task 3, after making a vertical incision in the kidney, it was placed on its side and longitudinally (Fig. 1i). During the training, two of the authors (HM and TA) were scopists, using a video system (VISERA Pro Video System Center OTV‐S7Pro; Olympus, Tokyo, Japan; Fig. 1a) and a 0° lens. All training sessions were video recorded for subsequent analyses. After the training session, completed questionnaires were collected including demographic data, experience of laparoscopic surgeries and simulation training. In Japan, in 2004, the ESSQ system was initiated, in which two double‐blinded referees evaluate an unedited surgical movie.^12^, ^13^ Information on this ESSQ qualification status was also collected. Regarding the similarity of porcine compared with human tissues and the educational merit, participants’ impressions were collected, for which a 5‐point Likert scale was used (tissue similarity, 5: very similar, 3: average, 1: very different, and educational merit, 5: very high, 3: average, 1: very low), and data from experts and intermediates were used for assessment. The subjective mental workload was also measured using the NASA‐TLX after each session. The NASA‐TLX contains six subscales: mental, physical and temporal task demands, effort, frustration, and perceived performance, and the questionnaire uses a 20‐point visual analog scale to measure the subjective mental workload based on the six aforementioned subscales.^14^

### Analysis

To assess the ability of our training model to grasp laparoscopic surgical skill levels of participants, two experts (TA and KH) evaluated the recorded footage according to GOALS in a blinded fashion.^15^

In the present study, we newly developed our original score sheet for each drill, named “ALL”, based on discussion among three of the authors (TA, KH and KM). Table 1 shows each domain of the ALL score. ALL scores were also evaluated in the same manner. To evaluate intraobserver reliability (the degree of agreement among assessments carried out by a single rater) of ALL scores, 55 training sessions for task 1, 49 training sessions for task 2, and 55 training sessions for task 3 were assessed twice by TA and HK.

**Table 1:** Assessment sheet of ALL

Tasks 1 and 2					
Domain	1	2	3	4	5
Traction	Usually not performed		Performed half of the time		Tissue is always stretched out under appropriate tension to visualize connective tissue surrounding the vessels
Blunt dissection	Usually not performed		Performed half of the time		Tissue is always dissected (blunt dissection) in a safe manner under direct visualization
Sharp dissection	Usually not performed		Performed half of the time		Tissue is always dissected (sharp dissection) in a safe manner under direct visualization
Skeltonization of vascular structure	Usually not performed		Performed half of the time		Vascular structure is always dissected sufficiently for subsequent ligation by Hem‐o‐lok (Weck‐lok)
Applying Hem‐o‐lok (Weck‐lok)	Usually not performed		Performed half of the time		Hem‐o‐lok (Weck‐lok) is always placed perpendicular to the vessel, and closed safely under direct visualization

Task 3					
Domain	1	2	3	4	5
Needle positioning 1	Usually not performed		Performed half of the time		Needle is always held at 1/2 to 2/3 from needle tip
Needle positioning 2	Usually not performed		Performed half of the time		Needle is always loaded perpendicular to needle driver
Needle entry	Usually not performed		Performed half of the time		Needle is always placed at 80–100‐degree angle to tissue
Needle driving and tissue trauma	Usually not performed		Performed half of the time		Needle is always removed with one movement along its curve
Knot tying	Usually not performed		Performed half of the time		Smoothly making an appropriate knot
Knot slipping	Usually not performed		Performed half of the time		Knot is smoothly slipped without tissue or thread damage

For both GOALS and ALL scores, the two raters’ mean scores were used for subsequent analyses. According to participants’ surgical experiences (experts: ≥50 laparoscopic surgeries, intermediates: 10–49, novices: 0–9), mean scores of GOALS, mean scores of ALL and NASA‐TLX scores at the time of their first training session were compared among the groups. In the present analyses, we chose a cut‐off of 50 cases to define the “expert” category based on a study reporting a shorter operative time on treating >50 cases,^16^ and an expert opinion.^17^ The ESSQ status was also used for comparison. ROC curve analysis was also used to assess the utility of each task to identify the ESSQ qualification status. In participants undergoing the present training multiple times, the changes of those scores were also evaluated.

The operative time (time to complete each task) were also calculated from the recorded footage (MH), and compared among the three groups. In task 3, 14 participants (13 novices, and one expert due to an emergent call) did not complete the three knots, and the crude time was used for the analysis.

### Statistical analysis

The Mann–Whitney *U*‐test or Kruskal–Wallis test was utilized to assess differences among groups. The ICC (2, 1) was evaluated for interrater reliability (the degree of agreement among raters) of GOALS and ALL scores. Regarding ALL scores, ICC (1, 1) was also calculated to evaluate intraobserver reliability (the degree of agreement among assessments carried out by a single rater). All statistical analyses were carried out using jmp 14 (SAS, Tokyo, Japan) or spss version 21 (IBM, Tokyo, Japan).

## Results

Table 2 shows a summary of participants’ backgrounds. A total of 32 urological doctors, eight young trainees and 14 medical students voluntarily joined the present training. A total of 13 participants were categorized as experts, eight as intermediates and 33 as novices. A total of 10 participants had the ESSQ qualification. Of the 54, 15 underwent the training multiple times (from two to six times), which resulted in a total of 80 sessions. Regarding task 2, it was cancelled in six sessions because of the poor condition of porcine organs. In addition, one session was accidentally not video recorded (one novice). Therefore, a total of 231 sessions (task 1: *n* = 79, task 2: *n* = 73, task 3: *n* = 79) were available for further analyses. Table 3 summarizes the interrater correlation of GOALS and ALL scores, and the intrarater correlation of ALL scores. Good interrater correlation was confirmed in both GOALS and ALL scores, and good intrarater correlation was also confirmed in ALL scores.

**Fig. 2:**
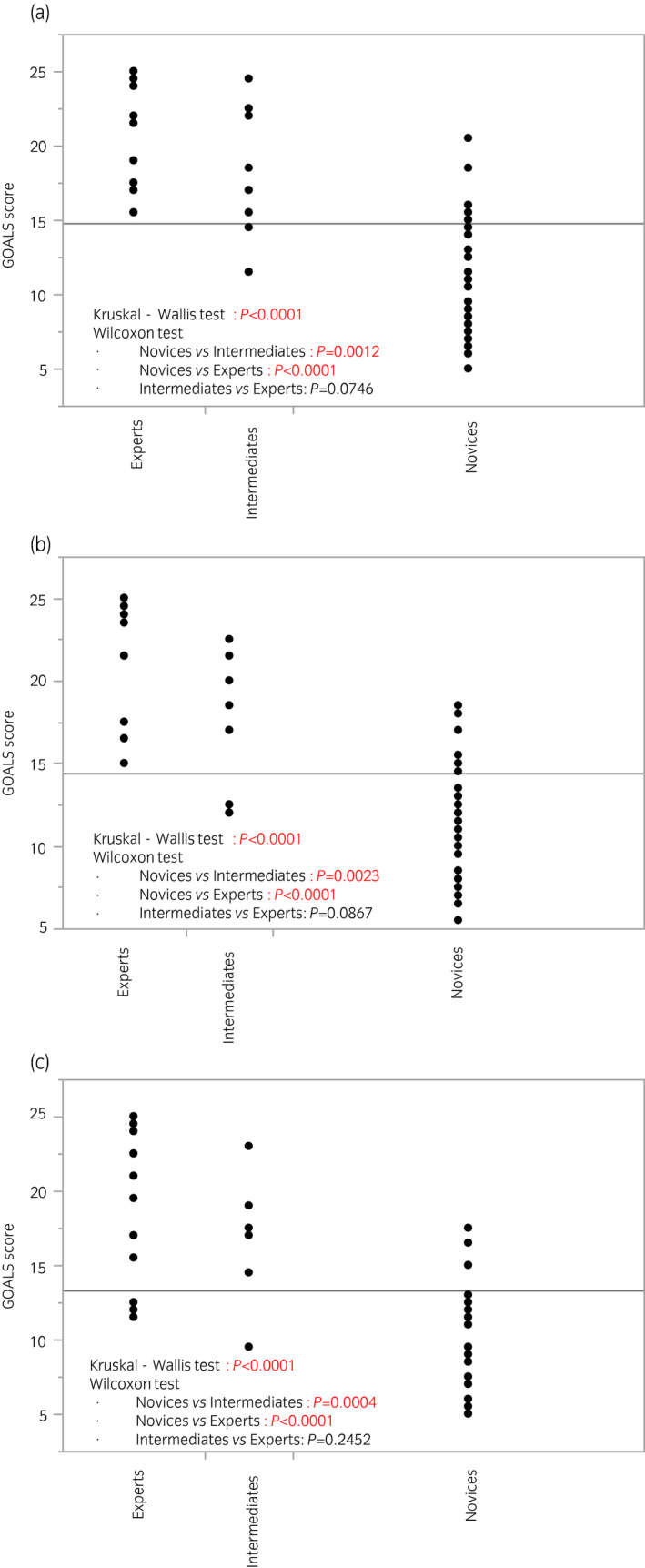
GOALS scores at the time of participants’ first training session divided by previous experience of laparoscopic surgery. (a) Task 1. (b) Task 2. (c) Task 3. There were significant differences in GOALS scores among the three groups in all three tasks.

**Fig. 3:**
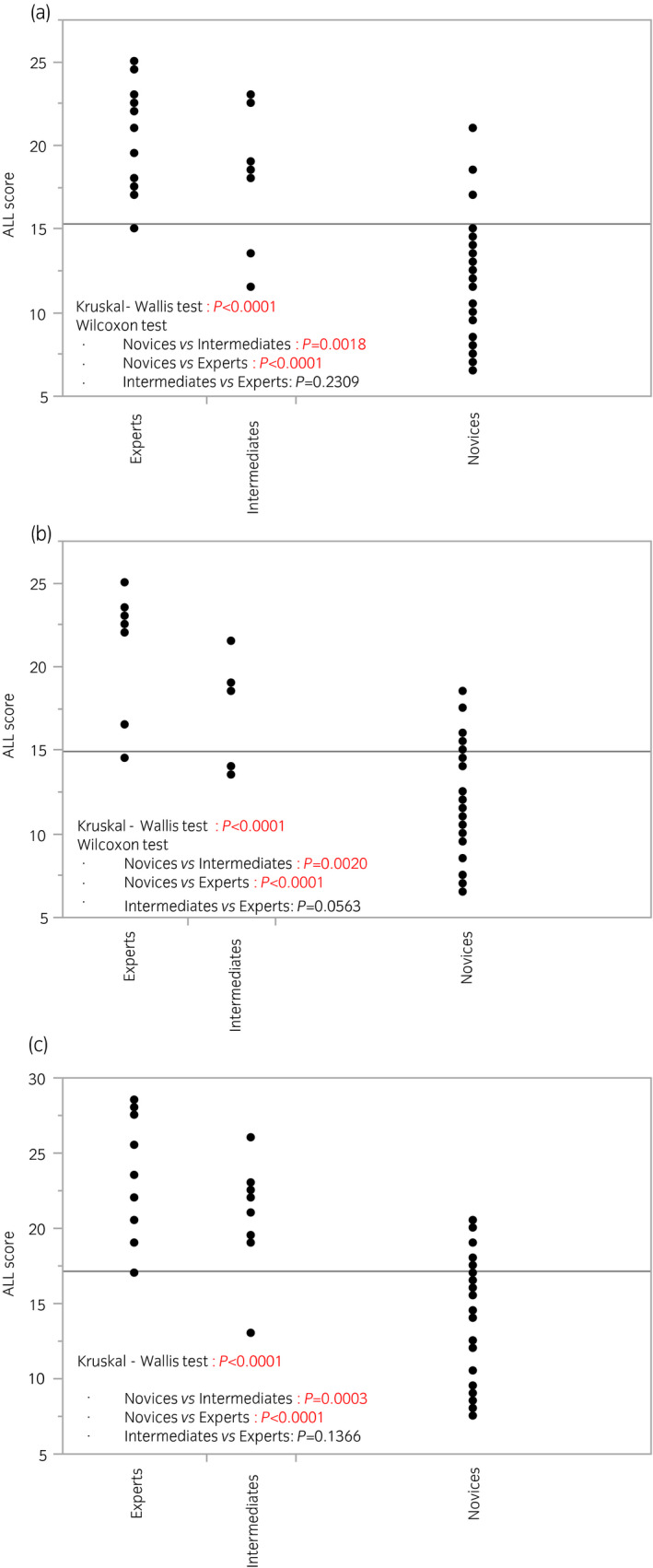
ALL scores at the time of participants’ first training session divided by previous experience of laparoscopic surgery. (a) Task 1. (b) Task 2. (c) Task 3. There were significant differences in ALL scores among the three groups in all three tasks.

Figures 2 and 3 show GOALS and ALL scores at the time of the participants’ first session, divided by previous surgical experiences. There were significant differences both in GOALS and ALL scores among the three groups in all three tasks, although the difference between experts and intermediates was marginal. Figure S1 shows a comparative study of operative times among the three groups. There were significant differences among the three groups in all three tasks, although the differences on two‐group comparison did not always remain significant.

**Table 2:** Summary of participants’ backgrounds

	*n* = 54
Age (years)	Median 29 (range 20–52)
Sex (male/female)	40/14
Background	
Urologists	*n* = 32
Medical students	*n* = 14
Junior residents	*n* = 8
Experience of laparoscopic surgery	
Experts (≥50 surgeries)	*n* = 13
Intermediates (10–49)	*n* = 8
Novices (0–9)	*n* = 33
Endoscopic surgical skill qualification (yes/no)	10/44
Experience of simulation training (yes/no)	36/18

**Table 3:** Summary of interrater reliability of GOALS and ALL scores, and intrarater reliability of ALL scores

	Task 1 (*n* = 79)	Task 2 (*n* = 73)	Task 3 (*n* = 79)
Interrater reliability†			
ICC (2, 1), GOALS score	0.745	0.718	0.857
ICC (2, 1), ALL score	0.692	0.693	0.844

	Task 1 (*n* = 55)	Task 2 (*n* = 49)	Task 3 (*n* = 55)
Intrarater reliability of ALL score‡			
ICC (1, 1), TA	0.786	0.824	0.864
ICC (1, 1), KH	0.9	0.881	0.941

† Interrater reliability is the degree of agreement among raters.

‡ Intra‐rater reliability is the degree of agreement among assessments carried out by a single rater.

**Fig. 4:**
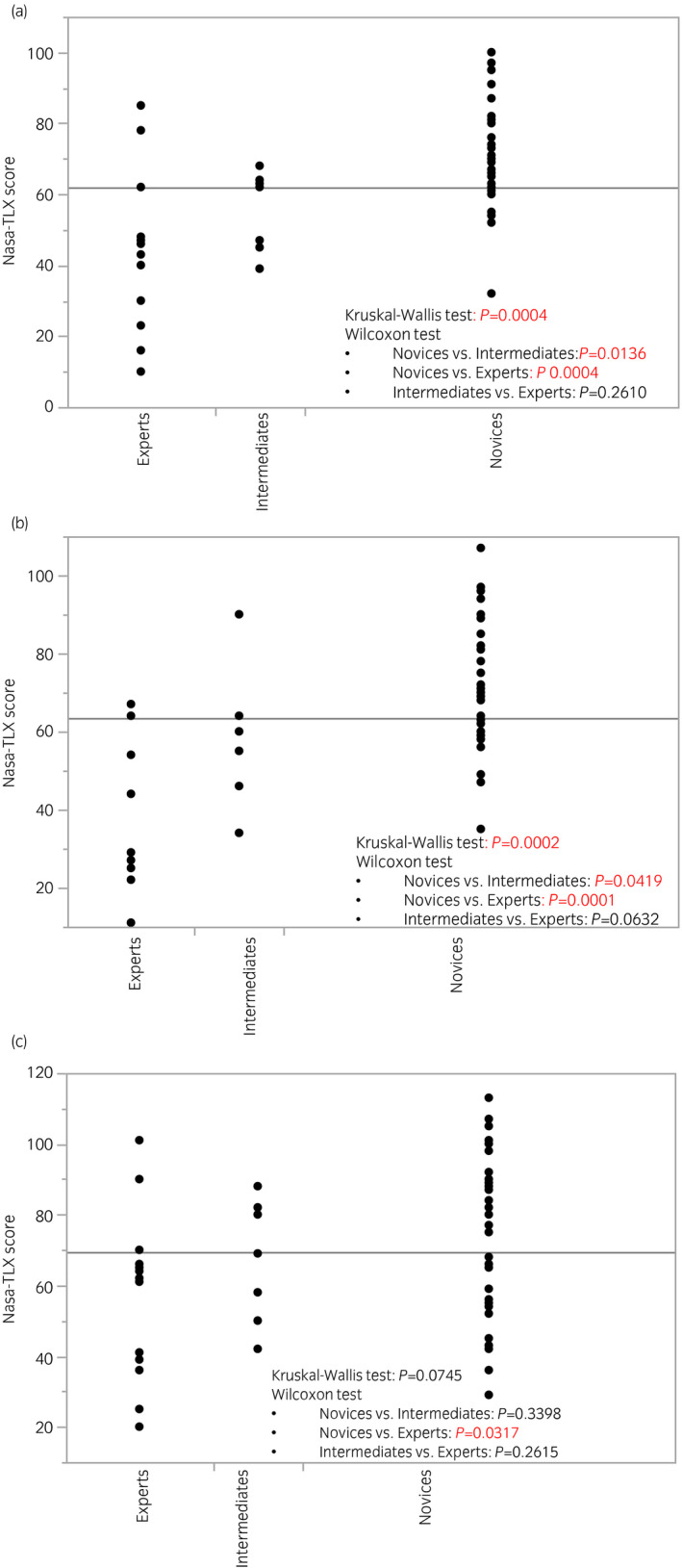
NASA‐TLX scores at the time of participants’ first training session divided by previous experience of laparoscopic surgery. (a) Task 1. (b) Task 2. (c) Task 3. Higher NASA‐TLX scores were observed in novices, and there were significant differences in tasks 1 (*P* = 0.0004) and 2 (*P* = 0.0002), and marginal differences in task 3 (*P* = 0.0745) among the three groups.

**Fig. 5:**
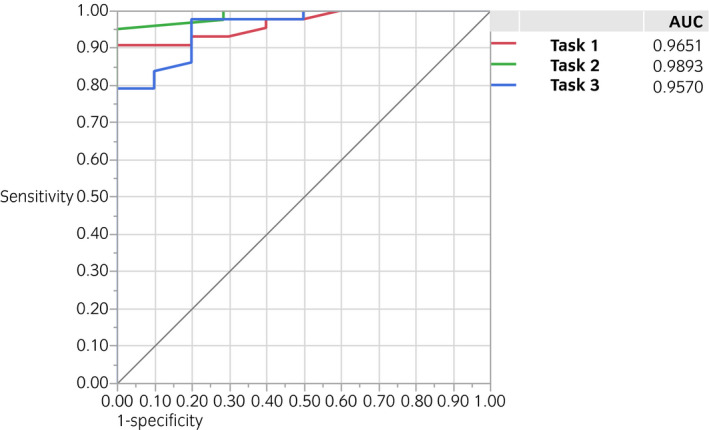
ROC curves of tasks 1, 2 and 3 for classifying the ESSQ qualification status based on GOALS score. All three tasks showed good separability.

Regarding NASA‐TLX scores, higher scores were observed in novices, and there were significant differences in tasks 1 (*P* = 0.0004) and 2 (*P* = 0.0002), and marginal differences in task 3 (*P* = 0.0745) among the three groups (Fig. 4). The comparative study between the ESSQ‐qualified surgeons and others also showed significant differences, whereby the ESSQ‐qualified surgeons showed higher GOALS/ALL scores and lower NASA‐TLX scores (Figs [Link], [Link], [Link]). Figure 5 shows the results of ROC analyses. The GOALS score could be used to identify the ESSQ qualification status, with a cut‐off value of 19, sensitivity of 100% and specificity of 90.7% in task 1, 20, 100% and 90.9%, respectively, in task 2, and 15, 100% and 74.4%, respectively, in task 3. ROC curves based on the ALL score also showed good utility to identify the status (Fig. S5).

**Fig. 6:**
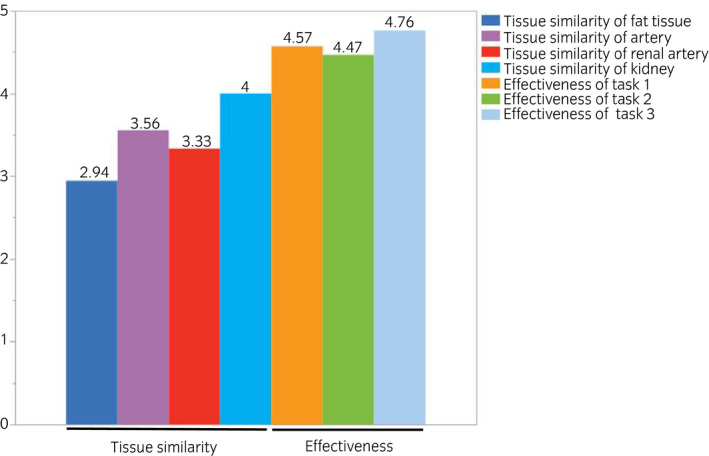
Tissue similarity and effectiveness of each task evaluated by the experts and intermediates. Most of the aspects were rated as above average, except for fat tissue. Both the intermediates and experts rated all three tasks as being effective for training.

**Fig. 7:**
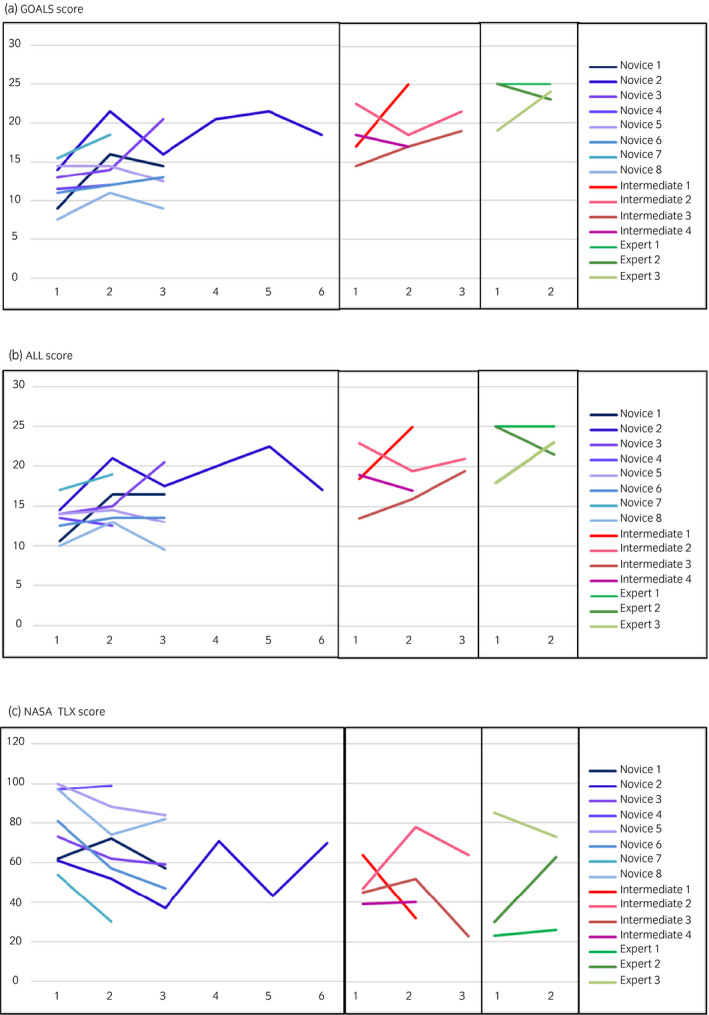
Learning curves of task 1 in the 15 participants who underwent the training multiple times. (a) GOALS score. (b) ALL score. (c) NASA‐TLX score. No constant trend was observed among the participants, although an increasing tendency in GOALS and ALL scores, and a decreasing tendency in NASA‐TLX scores were observed in several participants.

Figure 6 shows a summary of tissue similarity, as evaluated by experts and intermediates. Most of the aspects were rated as above average, except for fat tissue. The major opinion was that the fat tissue was harder than in humans. Regarding the usefulness of the present model, both the intermediates and experts rated all three tasks as being effective for training. Figure 7 shows the learning curves of task 1 for the 15 participants who participated in the training multiple times. No constant trend was observed among the participants, although an increasing tendency in GOALS and ALL scores, and a decreasing tendency in NASA‐TLX scores were observed in several participants. The same results were observed in tasks 2 and 3 (data not shown).

## Discussion

To help young trainees learn a broad range of techniques in laparoscopic surgery, such as grasping tissue, tissue traction and dissection, applying a Hem‐o‐lok clip, and suturing/knotting, we started the present wet lab training using the three aforementioned tasks. Cadaveric animal organ training models have been utilized in laparoscopic surgical training for decades, and several models have been reported, simulating relatively complex laparoscopic surgical procedures. Ramachandran *et al*. developed a model for laparoscopic pyeloplasty training using a chicken crop and esophagus, and observed skill improvement in the three trainees during the simulation training.^18^ Boza *et al*. reported a cadaveric porcine model (a side‐to‐side stapled jejuno‐jejunostomy) for assessment of laparoscopic Roux‐en‐Y gastric bypass.^19^ Using a motion tracking device, they observed that their cadaveric model showed concurrent validity with significant correlations between performance using the organ model and on treating patients regarding dexterity measures of eight surgeons with various levels of experience. Recently, Liu *et al*. reported a novel laparoscopic simulation model involving a porcine liver that was continuously perfused, and observed skill improvements in laparoscopic cholecystectomy in 43 participants during their simulation training.^20^ Compared with these cadaveric models, we consider our model to be a generic training model for young trainees to learn a broad range of essential skills in laparoscopic surgery, including grasping tissue, tissue traction and dissection, applying a Hem‐o‐lok clip, and suturing/knotting, as aforementioned. To our knowledge, the swine aorta model (task 1) is a novel laparoscopic training model for tissue dissection around vessels. Regarding the six cancelled sessions of task 2, this was because the vascular pedicles of the renal hilum had already been removed by the meat processing wholesaler, but this was easily resolved after the explanation of our training aim to that wholesaler.

To evaluate the ability to differentiate skill levels of participants (construct validity), we compared GOALS and ALL scores among the novices, intermediates, and experts, and observed the following results. First, ICCs regarding inter‐ and intrarater reliability showed that both GOALS and ALL scores were reliable indexes to assess essential laparoscopic skills. Second, there were significant differences in GOALS and ALL scores among the three groups. As shown in Figures 2 and 3, there were significant differences between novices and intermediates or experts, whereas there were marginal differences between intermediates and experts. We consider that these observations are reasonable, because our training model is designed for novices to learn essential laparoscopic surgical skills, and case numbers would not always reflect the present surgical skills after dozens of experiences. As shown in Figure 5, the GOALS score could be used to effectively identify the ESSQ qualification status in all three tasks. We consider that the cut‐off values shown in the ROC analysis could be used as benchmark scores, and novices will need to repeat training in the laboratory until competency is achieved.

Regarding the ALL score, we originally developed it because we expected that the procedure‐based assessment could better differentiate the skill level of participants than GOALS (generic assessment method). As described above, both GOALS and ALL scores well‐differentiated the skill level of participants, and we observed very high correlations between the ALL and GOALS scores (task 1: *r* = 0.986, task 2: *r* = 0.979, task 3: *r* = 0.981, data not shown). We now consider that the GOALS score is very useful to differentiate the skill level, and the ALL score might have added value to provide more concrete feedback to trainees.

NASA‐TLX is a subjective assessment tool of workload based on six weighted subscales: mental demand, physical demand, temporal demand, performance, effort and frustration level, and has been widely utilized in a variety of fields, including medicine.^21^, ^22^, ^23^ Our group also reported that under repeated simulation training in ureteroscopy in a mock operating theater, participants showed a continuous decrease in the mental workload, whereas improvements in the technical skills reached a plateau over the six sessions.^24^ In the present study, although significant variability in NASA‐TLX scores existed within each group, the scores were higher in the novice group (Fig. 4), and a downward trend was observed in several participants with multiple training sessions (Fig. 7). Because an excessive cognitive workload might increase fatigue and have a negative impact on surgical performance, assessing the NASA‐TLX score during simulation training might help educators to decide whether trainees are ready to tackle a real surgical case from the perspective of the cognitive workload.

We recognize that the present study had several limitations, such as the small sample size and lack of sample size calculation. We do not have sufficient data regarding the learning curves on repeating the present wet lab training, data regarding skill retention after training or subsequent skill improvement in real clinical practice (predictive validity). Regarding the running cost of disposable parts, a swine aorta costs approximately $7, a swine kidney approximately $7, one set of Hem‐o‐lok clips approximately $36 and one 2‐0 CT‐1 needle approximately $6, and our model requires additional resources, including surgical instruments, a training room and manpower. The VR simulator might be a superior modality, when not considering the initial high cost. Recently, our group reported good face, content and construct validity for the LapVision nephrectomy module, a newly developed VR simulator.^25^ However, we feel that more realistic computer graphics and haptic feedback are still necessary. As described in the Introduction, residents preferred live animals or animal tissue wet lab as simulation tools, rather than the VR simulator.^10^, ^11^ Compared with live swine surgical training, our cadaveric organ model is much simpler, more economical and ideal in terms of animal life protection. However, lack of bleeding is one of the weakest points, which probably makes our wet lab training environment less stressful. Live animal surgical training can provide the most realistic surgical situation, and trainees are expected to tackle difficult situations, such as unexpected bleeding. In addition, many surgeons now use bipolar vessel sealers during laparoscopic surgeries, and live animal surgical training might be ideal for bipolar vessel sealers and bleeding control. Because VR simulators and live animal surgical training have different benefits than cadaveric organ trainings, educators should develop an integrated laparoscopic surgical training program using different training modalities.

In conclusion, we observed good construct validity of our cadaveric porcine organ model. Differences in the NASA‐TLX score were also observed according to previous laparoscopic surgical experiences. These results show the ability of this model to assess both laparoscopic surgical skills and mental workloads, which could help educators comprehend trainees’ level of achievement outside the operating theater. Given the decreasing opportunity to carry out pure laparoscopic surgeries due to the rapid dissemination of robot‐assisted surgery, simulation training is becoming more important to learn key skills of laparoscopic surgery. We believe that our model can offer a practical training opportunity outside the operating theater. Future study is necessary to confirm its educational merit.

## Conflict of interest

None declared.

## Supporting information


**Figure S1**. Operative time in participants’ first training session divided by previous experience of laparoscopic surgery. (a) Task 1. (b) Task 2. (c) Task 3.

**Figure S2**. GOALS scores at the time of participants’ first training session divided by the ESSQ qualification status. (a) Task 1. (b) Task 2. (c) Task 3.

**Figure S3**. ALL scores at the time of participants’ first training session divided by the ESSQ qualification status. (a) Task 1. (b) Task 2. (c) Task 3.

**Figure S4**. NASA‐TLX scores at the time of participants’ first training session divided by the ESSQ qualification status. (a) Task 1. (b) Task 2. (c) Task 3.

**Figure S5**. ROC curves of tasks 1, 2 and 3 for classifying the ESSQ qualification status based on ALL scores.

**Video S1**. Edited movie of task 1.
