# Supplementary material for: Development and validation of a porcine organ model for training in essential laparoscopic surgical skills
Source: Int J Urol. 2020 Aug 3;27(10):929–38. doi: 10.1111/iju.14315 (PMC7589398; doi:10.1111/iju.14315)
Supplement: Supplementary file 1 — Figure S1. Operative time in participants’ first training session divided by previous experience of laparoscopic surgery. (a) Task 1. (b) Task 2. (c) Task 3. [file IJU-27-929-s001.pptx]

## Slide 1
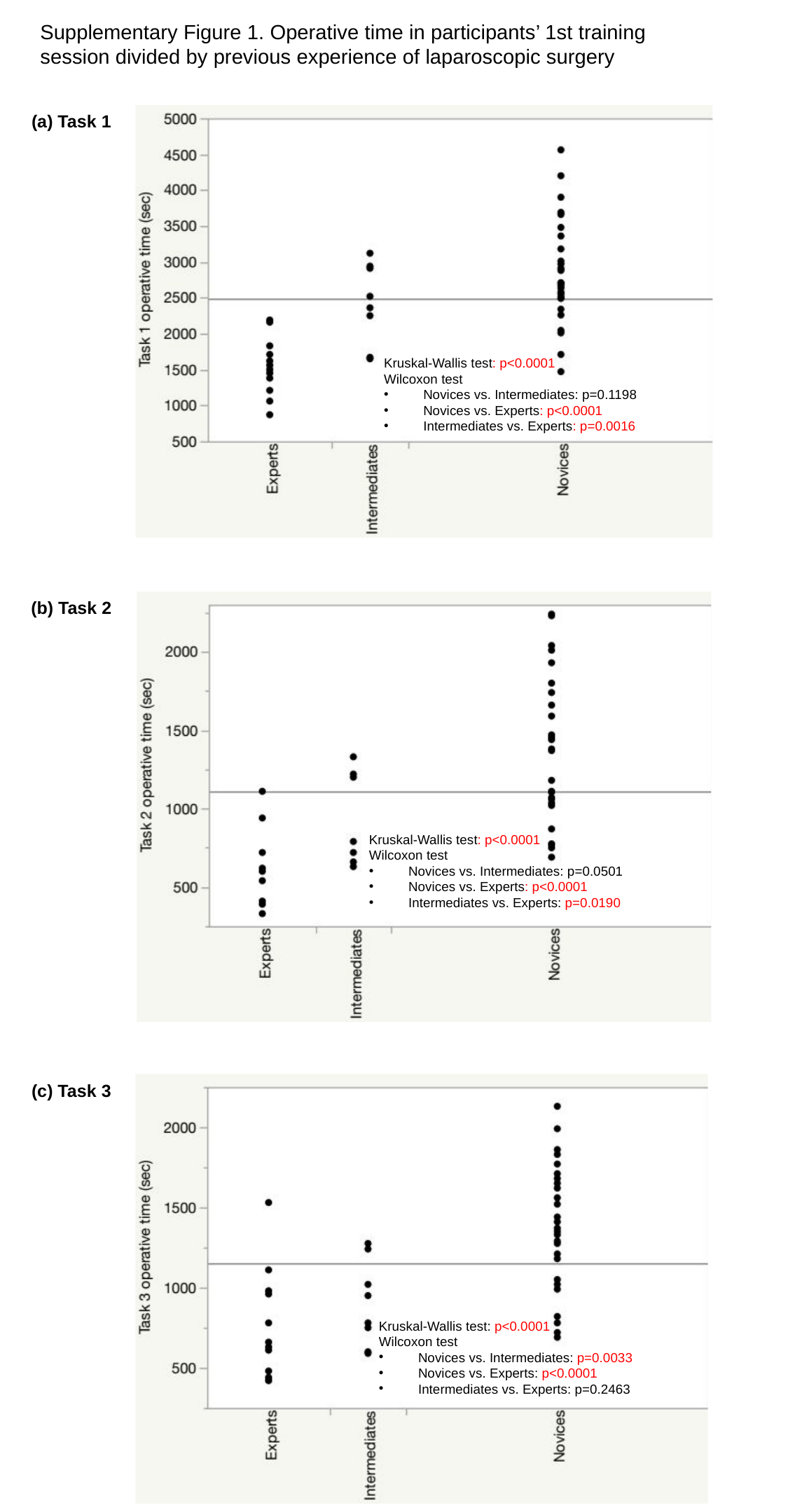

Supplementary Figure 1. Operative time in participants’ 1st training session divided by previous experience of laparoscopic surgery
(a) Task 1
Kruskal-Wallis test: p<0.0001
Wilcoxon test
Novices vs. Intermediates: p=0.1198
Novices vs. Experts: p<0.0001
Intermediates vs. Experts: p=0.0016
(b) Task 2
Kruskal-Wallis test: p<0.0001
Wilcoxon test
Novices vs. Intermediates: p=0.0501
Novices vs. Experts: p<0.0001
Intermediates vs. Experts: p=0.0190
(c) Task 3
Kruskal-Wallis test: p<0.0001
Wilcoxon test
Novices vs. Intermediates: p=0.0033
Novices vs. Experts: p<0.0001
Intermediates vs. Experts: p=0.2463
