# Supplementary material for: Development and validation of a porcine organ model for training in essential laparoscopic surgical skills
Source: Int J Urol. 2020 Aug 3;27(10):929–38. doi: 10.1111/iju.14315 (PMC7589398; doi:10.1111/iju.14315)
Supplement: Supplementary file 4 — Figure S4. NASA‐TLX scores at the time of participants’ first training session divided by the ESSQ qualification status. (a) Task 1. (b) Task 2. (c) Task 3. [file IJU-27-929-s004.pptx]

## Slide 1
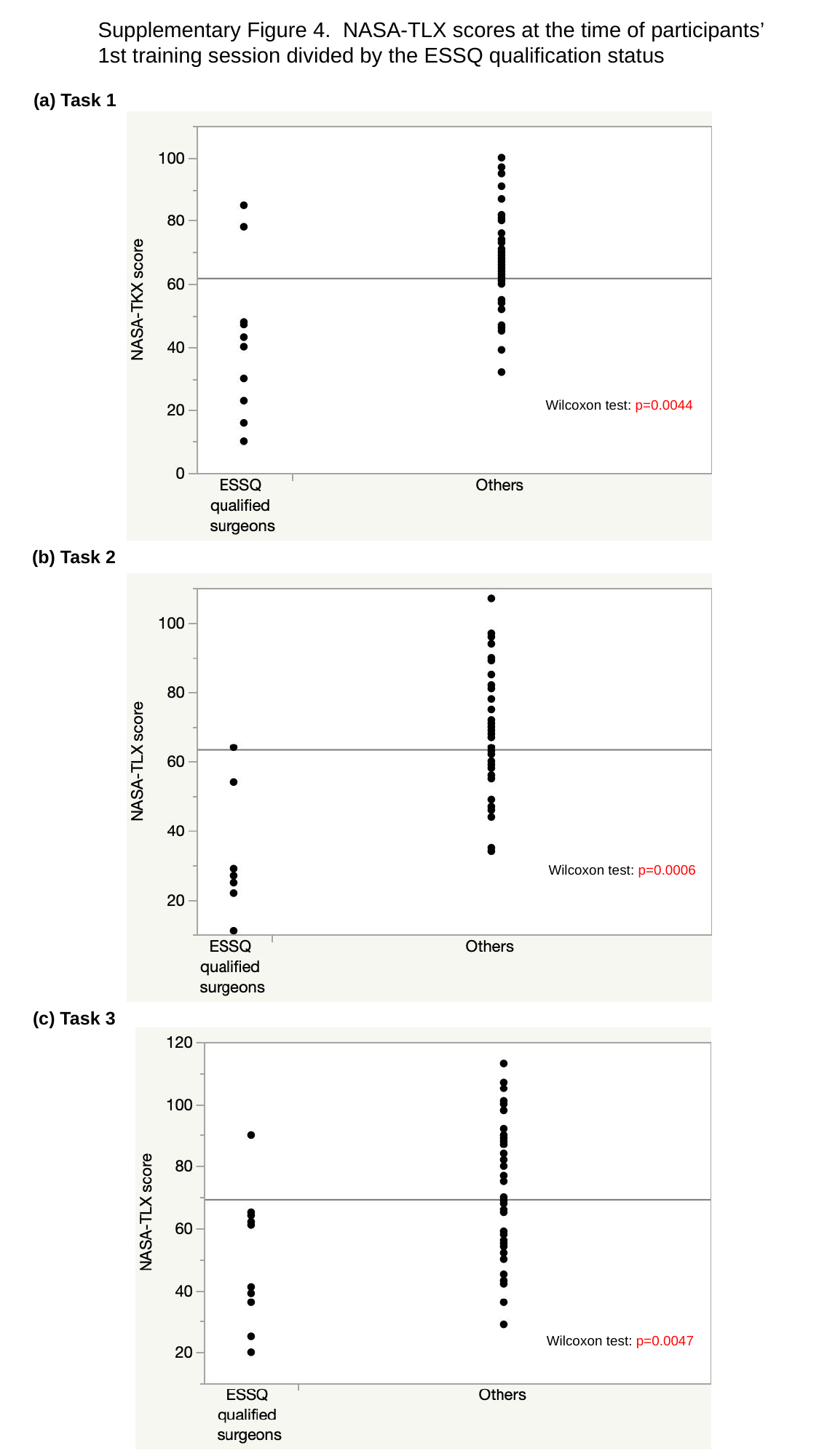

Supplementary Figure 4. NASA-TLX scores at the time of participants’ 1st training session divided by the ESSQ qualification status
(a) Task 1
Wilcoxon test: p=0.0044
(b) Task 2
Wilcoxon test: p=0.0006
(c) Task 3
Wilcoxon test: p=0.0047
