# Supplementary material for: Development and validation of a porcine organ model for training in essential laparoscopic surgical skills
Source: Int J Urol. 2020 Aug 3;27(10):929–38. doi: 10.1111/iju.14315 (PMC7589398; doi:10.1111/iju.14315)
Supplement: Supplementary file 5 — Figure S5. ROC curves of tasks 1, 2 and 3 for classifying the ESSQ qualification status based on ALL scores. [file IJU-27-929-s005.pptx]

## Slide 1
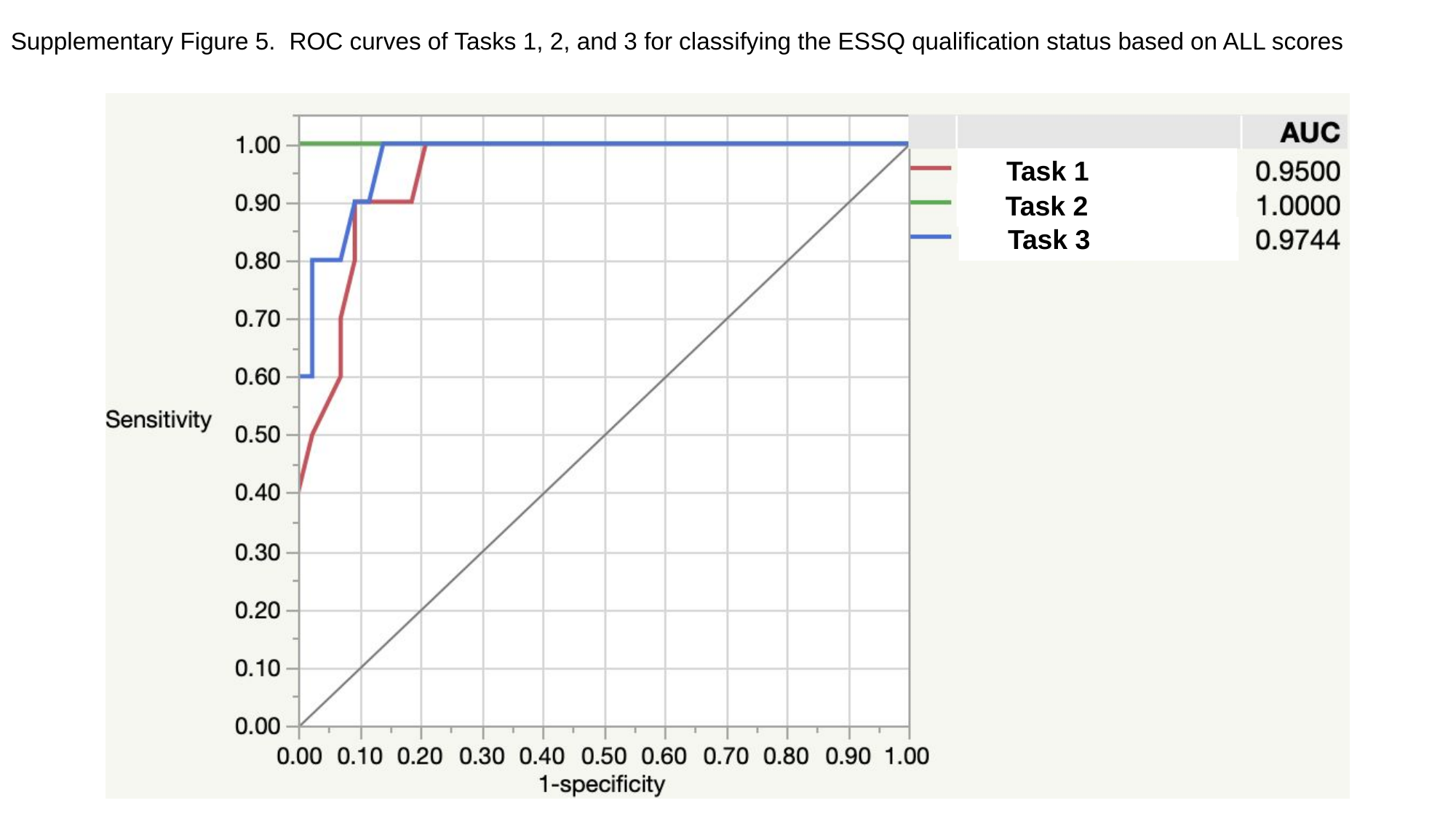

Supplementary Figure 5. ROC curves of Tasks 1, 2, and 3 for classifying the ESSQ qualification status based on ALL scores
 Task 1
 Task 2
 Task 3
